# Toward New Transmission-Blocking Combination Therapies: Pharmacokinetics of 10-Amino-Artemisinins and 11-Aza-Artemisinin and Comparison with Dihydroartemisinin and Artemether

**DOI:** 10.1128/AAC.00990-21

**Published:** 2021-07-16

**Authors:** Daniel J. Watson, Lizahn Laing, Liezl Gibhard, Ho Ning Wong, Richard K. Haynes, Lubbe Wiesner

**Affiliations:** a Division of Clinical Pharmacology, Department of Medicine, University of Cape Town, Cape Town, South Africa; b H3D, Department of Chemistry, University of Cape Town, Cape Town, South Africa; c Centre of Excellence for Pharmaceutical Sciences, Faculty of Health Sciences, North-West University, Potchefstroom, South Africa

**Keywords:** amino-artemisinins, pharmacokinetics, antimalarial agents, combination therapies, transmission-blocking

## Abstract

As artemisinin combination therapies (ACTs) are compromised by resistance, we are evaluating triple combination therapies (TACTs) comprising an amino-artemisinin, a redox drug, and a third drug with a different mode of action. Thus, here we briefly review efficacy data on artemisone, artemiside, other amino-artemisinins, and 11-aza-artemisinin and conduct absorption, distribution, and metabolism and excretion (ADME) profiling *in vitro* and pharmacokinetic (PK) profiling *in vivo* via intravenous (i.v.) and oral (p.o.) administration to mice. The sulfamide derivative has a notably long murine microsomal half-life (*t*_1/2_ > 150 min), low intrinsic liver clearance and total plasma clearance rates (CL_int_ 189.4, CL_tot_ 32.2 ml/min/kg), and high relative bioavailability (*F* = 59%). Kinetics are somewhat similar for 11-aza-artemisinin (*t*_1/2_ > 150 min, CL_int_ = 576.9, CL_tot_ = 75.0 ml/min/kg), although bioavailability is lower (*F* = 14%). In contrast, artemether is rapidly metabolized to dihydroartemisinin (DHA) (*t*_1/2_ = 17.4 min) and eliminated (CL_int_ = 855.0, CL_tot_ = 119.7 ml/min/kg) and has low oral bioavailability (*F*) of 2%. While artemisone displays low *t*_1/2_ of <10 min and high CL_int_ of 302.1, it displays a low CL_tot_ of 42.3 ml/min/kg and moderate bioavailability (*F*) of 32%. Its active metabolite M1 displays a much-improved *t*_1/2_ of >150 min and a reduced CL_int_ of 37.4 ml/min/kg. Artemiside has *t*_1/2_ of 12.4 min, CL_int_ of 673.9, and CL_tot_ of 129.7 ml/kg/min, likely a reflection of its surprisingly rapid metabolism to artemisone, reported here for the first time. DHA is not formed from any amino-artemisinin. Overall, the efficacy and PK data strongly support the development of selected amino-artemisinins as components of new TACTs.

## TEXT

**FIG 1:**
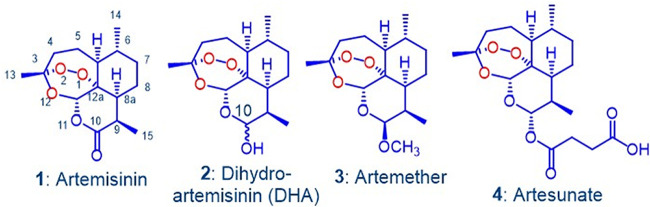
Artemisinin 1 and clinical derivatives DHA 2, artemether 3, and artesunate 4. The latter two are rapidly converted into DHA *in vivo* via metabolism or facile hydrolysis, respectively. As a hemiacetal, DHA rearranges irreversibly under physiological conditions into an active peroxyhemiacetal that in turn rearranges to the inert deoxyartemisinin (see references 41–44).

The efficacies of artemisinin combination therapies (ACTs) comprising one of dihydroartemisinin (DHA) 2, artemether 3, or artesunate 4 (Fig. 1) with piperaquine, mefloquine, amodiaquine, lumefantrine, or other are now being degraded in the face of increasing resistance of Plasmodium falciparum to the artemisinin component (1–3). Although initially reported in the countries of the greater Mekong subregion in Southeast Asia, evolution of resistant strains in other countries is now being reported (3–5). Resistance arises via induction of ring-stage quiescence in parasite phenotypes carrying point mutations, including C580Y in the P. falciparum Kelch 13 propeller domain (*Pf*K13) (6–11). It is held that DHA, used as a drug in its own right or as the principal metabolite of artemether and artesunate, competitively binds to *Pf*PI3K, which prevents the kinase binding to *Pf*K13. This thereby inhibits ubiquitinylation and proteasome degradation leading to cell cycle arrest and quiescence (12, 13). The problem is now compounded by decreasing efficacy of the ACT partner drug (2, 14–17). The response has been to extend the treatment regimen of the one ACT, to use two different ACTs in sequence (18, 19), or to conceptualize use of triple combination therapy (TACT) (14, 17). In the last case, DHA-piperaquine used with mefloquine or artemether-lumefantrine with amodiaquine in clinical trials indeed resulted in enhanced efficacy; for the DHA-piperaquine-mefloquine regimen, efficacy was improved from 48% for the ACT to 98% for the TACT (20). However, use of such TACTs (as is the case with current ACTs) involving the current clinical artemisinins combined with known drugs without any regard to intrinsic mechanism (21) or pharmacodynamic or pharmacokinetic aspects also carries the risk of amplifying resistance (22, 23). Also, mass drug administration (MDA) programs using ACTs to clear subclinical reservoirs of resistant parasites in Southeast Asia are under way. Thus, administration of 3-day courses of DHA-piperaquine plus single dose primaquine each month for 3 months to village populations in Cambodia is being carried out (24, 25). The practice has been criticized on ethical grounds, ambiguity in definition of subclinical parasites, and use of substantial resources in the face of outcomes that are not easily defined (26–28). Irrespective of these issues, the abnormally protracted treatment and MDA regimens do require increased pharmacovigilance (29). Nevertheless, other control modalities, including enhanced surveillance, reliable detection and treatment of infected individuals from urban, agricultural, or remote forested areas, use of insecticide-treated bednets, vector control, etc., are enhancing progress toward the goal of achieving malaria elimination in the greater Mekong subregion (30).

**FIG 2:**
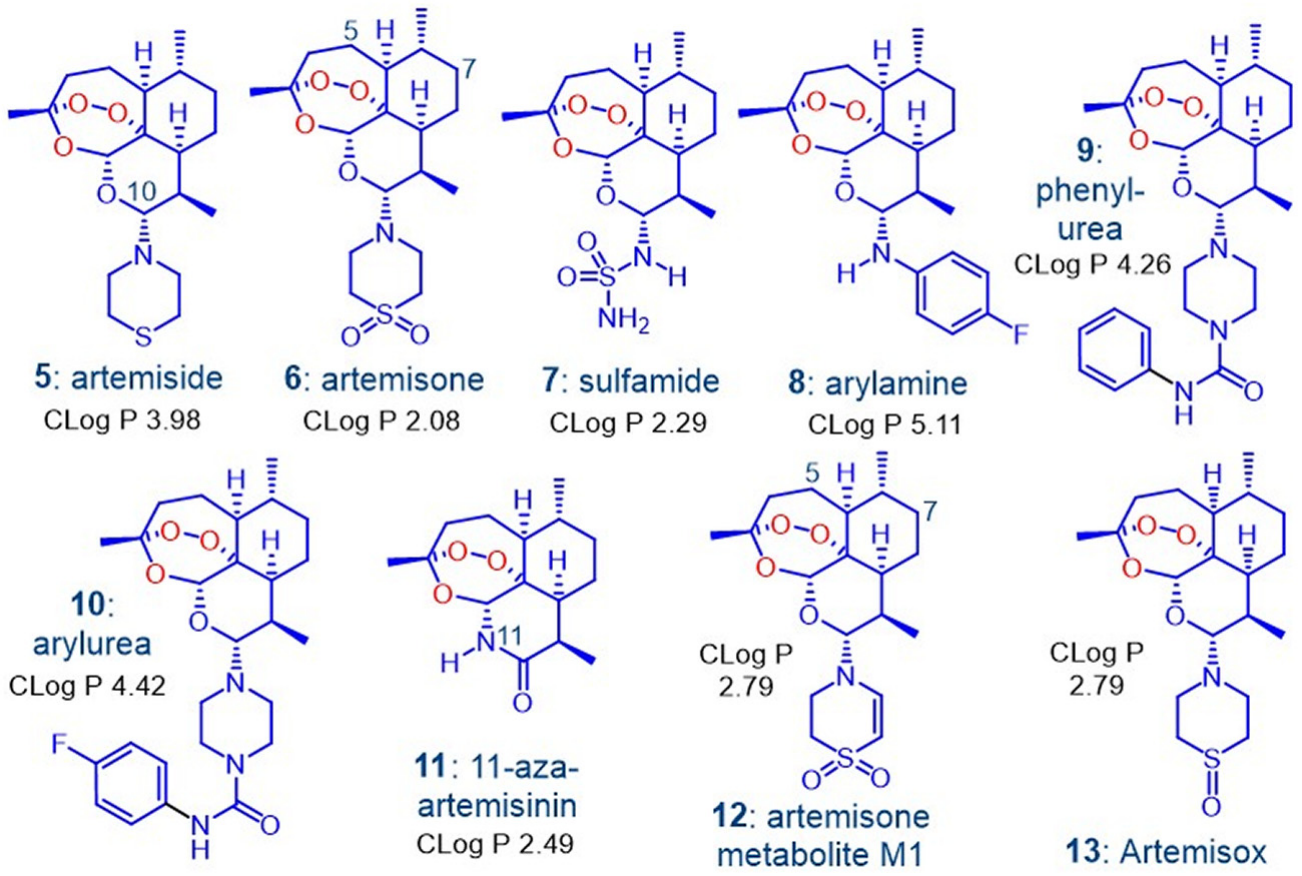
The amino-artemisinins 5 to 10 in which the exocyclic oxygen atom at C-10 of the clinical artemisinins (Fig. 1) is replaced by a nitrogen atom and 11-aza-artemisinin 11 in which an –NH group replaces O-11. As discussed below, artemiside 5 is rapidly metabolized to artemisone 6, which must proceed via artemisox 13, and then to M1 12, the primary metabolite of artemisone. Calculated log *P* data are given, and efficacy data for 1 to 12 appear in references 47, 48, and 61–63 and are summarized in Tables S1 to S7.

The artemisinins in the current ACTs (Fig. 1) have been in use since their introduction by the Chinese in the 1970s and 1980s (31–33). These, including DHA, are inducers of their own metabolism (34–36) and elicit neurotoxicity concerns (29, 37–40). DHA is thermally unstable and in aqueous solution readily rearranges to a peroxyhemiacetal (41–45) whose formation also may intrude during preparation and storage of DHA (45). Such properties highlight the intemperance associated with development of DHA as an antimalarial drug (42, 43, 46). Overall, new therapies involving rational combinations of novel drugs are urgently required for treatment of multidrug-resistant malaria (23). To this end, we are developing combinations based on discrete consideration of mechanism of action of the components (47, 48). For the first drug, we focus on new artemisinin derivatives that have enhanced efficacies against all blood stages of the malaria parasite and ideally retain baseline activities against artemisinin-resistant strains. The rationale and data supporting use of a redox-active second drug such as methylene blue which displays synergism with the new artemisinins (47, 48) and a third drug type such as a quinolone (49) based on a distinct target are presented in detail elsewhere. The artemisinin derivatives bear an amino group attached to C-10 (44) wherein antimalarial activity is enhanced (50). Examples are given in Fig. 2; these are structurally distinct to the artemisinins of Fig. 1 bearing an oxygen-linked substituent at C-10.

The best known amino-artemisinin is artemisone 6 (Fig. 2) (51, 52). It is nonneurotoxic (51, 53) and does not induce its own metabolism (54). The principal metabolite M1 12 (Fig. 2) is formed together with other metabolites structurally related to artemisone and M1 bearing hydroxyl groups at C-5 and C-7, all of which retain antimalarial activity (51, 54). Artemisone is active against murine cerebral malaria; treatment at prescribed low dose levels were completely curative, whereas at the same dose levels, DHA and artesunate, the latter of which is currently used for treatment of cerebral malaria, elicited no cure (55). In a phase IIa trial of patients with nonsevere malaria, artemisone was curative at one-third the dose level of artesunate (54, 56–59). With this earlier data serving as a backdrop to our current efforts, we needed to establish if other amino-artemisinins possess efficacy and drug metabolism and pharmacokinetic (DMPK) profiles superior to those of artemisone 6. Thus, we conducted efficacy screens on artemiside 5, the sulfide precursor of artemisone 6 (51, 52), on artemisone 6 itself as comparator, the 10-sulfamide 7 (51), the 10-(*p*-fluorophenyl)amino derivative 8 (60), the 10-(phenylpiperazine)- and 10-(*p*-fluorophenylpiperazine) urea derivatives 9 (47) and 10 (48), 11-aza-artemisinin 11 (61, 62), and the metabolite M1 (63) 12 (Fig. 2). Several were screened against asexual blood stages of artemisinin-resistant P. falciparum clones ARC08-22 (4G) and PL08-009 (5C) carrying the *Pf*KI3 C580Y mutation (64) and also against liver-stage P. berghei (*Pb*) sporozoites (48). The data are summarized in Tables S1 to S7 in the supplemental material.

Salient features are as follows. Artemiside, artemisone, and the arylurea possess 50% inhibitory concentration (IC_50_) activities ranging from 0.85 to 1.75 nM against asexual blood stages of CQ-sensitive *Pf*NF54 and multidrug-resistant K1 and W2 strains. The sulfamide, arylamine, and phenylurea are slightly less active against all strains (1.3 to 4.7 nM), and 11-aza-artemisinin is the least active (6.02 to 10.48 nM) (Table S2) (62). Cytotoxicity assays, including those against Chinese hamster ovary (CHO) cells, indicate that the arylamine and phenylurea (50% effective concentration [EC_50_] 2.4 to 2.9 μM) are more toxic than artemiside, artemisone, and the arylurea (204 to >271 μM) (Table S1) (47, 48). The amino-artemisinins are active against blood-stage *P. falciparum* gametocytes, with activities (IC_50_) against early-stage gametocytes (EG > 90% stage I to III) ranging from 1.94 nM for artemisone to 83 nM for the phenylurea. Against late-stage gametocytes (LG > 90% stage IV and V), the sulfamide is the least active (IC_50_ 419.4 nM), with activities of the other compounds ranging from 1.5 to 42.4 nM. Thus, the latter are appreciably more active than methylene blue, a known gametocytocidal agent (65), with EG IC_50_ of 95 nM and LG of 143 nM (Table S3). These activities thus portend utility of the amino-artemisinins as transmission-blocking agents (47, 48). 11-Aza-artemisinin is less active with EG IC_50_ of 170.4 and LG of 166.1 nM (Table S4) (62). Against liver-stage P. berghei sporozoites, IC_50_ values range from 28.3 nM for artemisone to 81.3 for artemiside to 105.5 nM for the arylurea (Table S5); in comparison, artemether has an IC_50_ of >10^4^ nM (48). Against asexual blood stages of artemisinin-resistant clones carrying the *Pf*KI3 C580Y mutation, artemiside and artemisone (IC_50_ W2 equal to 1.69 to 2.21 nM) are equipotent against clone ARC08-22 (4G) (1.62 to 2.43 nM) and more active against the PL08-009 (5C) clone (0.27 to 0.29 nM). The phenylurea is also active against the ARC08-22 (4G) clone. DHA elicits activities of 6.41 to 6.68 nM against these clones (Table S6) (48). The retention of efficacy of artemisone against artemisinin-resistant clones is consistent with earlier results involving Cambodian P. falciparum wild-type (WT) isolates transfected to strains CamWTC580Y (C580Y K13 mutation), Cam3.IIC580Y (C580Y K13), and Cam3.IIRev (wild-type K13 sequence), wherein it essentially retains baseline activities across all strains, as reflected in a mean IC_50_ 2.4 nM (66); activity against W2 is 1.9 nM. For DHA, activities are 11.2 and 5.2 nM, respectively. The metabolite M1 is active against drug-sensitive and- resistant strains of P. falciparum (IC_50_ 2.5 to 8.6 nM) but less so than artemisone (IC_50_ 0.7 to 1.1 nM) (63). However, artemisone and M1 do induce quiescence in W2 ring-stage parasites but not in a concentration-dependent manner; cultures treated with different concentrations of these drugs reach 1% parasitemia within the same time period (63). Work is under way probing the basis of this effect and how these and other drugs to be used in the putative combinations may modulate induction of quiescence in ring-stage assays involving the resistant phenotypes or indeed kill the quiescent rings.

Overall, artemiside, artemisone, and the arylurea are the frontrunners in terms of global efficacies and relatively low toxicities *in vitro*. However, expression of therapeutic efficacy depends not only on biological activity but also on pharmacokinetic (PK) properties. This becomes important for the envisaged drug combinations (67). We report here on the *in vitro* absorption, distribution, metabolism, and excretion (ADME) properties and PK measurement in a murine model of the compounds of Fig. 2. Initially, the arylamine 8 was also included, but preliminary PK assessment in the murine model unexpectedly indicated untoward toxicity, so it was not further examined. The data are directly compared to those of DHA and artemether. An evaluation of the compounds to be taken forward is then presented.

## RESULTS

### ADME profiling *in vitro*.

The kinetic solubilities, plasma protein binding, metabolic stability, and calculated intrinsic clearance rates and hepatic extraction ratio *E_H_* for mouse, rat, and human liver microsomes (68) are shown in Table 1.

**TABLE 1:** Solubilities, microsomal stabilities, and plasma protein binding of the artemisinins^*a*^

Compound	Solubility (μM)^*b*^			Microsomal half-life *t*_1/2_ (min)			Intrinsic liver clearance rate CL_int_ (ml/min/kg)			Hepatic extraction ratio *E_H_*			Plasma protein binding f_u_
	pH 2	FaSSIF pH 6.5^*c*^	pH 7.4	Mouse	Rat	Human	Mouse	Rat	Human	Mouse	Rat	Human	
DHA 2	<5^*d*^	<5^*d*^	<5^*d*^	135.9	90.0	51.7	50.2	34.7	41.5	0.4	0.4	0.7	ND^*e*^
Artemether 3	ND	ND	ND	17.4	<10	12.5	392.8	988.2	171.7	0.8	0.9	0.9	0.14
Artemiside 5	12.4	<5	<5	12.4	<10	<10	551.3	1,389.2	450.8	0.9	1.0	1.0	0.23
Artemisone 6	73.7	74.4	120.4	<10	<10	<10	805.2	342.5	242.6	0.9	0.9	0.9	0.14
Sulfamide 7	>150	<5	11.5	>150	94.9	>150	41.3	32.9	5.6	0.3	0.4	0.2	0.17
Phenylurea 9	75.6	<5	<5	13.5	<10	<10	506.0	1,539.0	394.5	1.7	1.0	ND	0.14
Arylurea 10	10.0	24.0	27.0	<10	ND	<10	2,485.0	ND	531.0	0.6	ND	0.6	0.02
11-Aza-artemisinin 11	>150^*f*^	>150^*f*^	144.2^*f*^	>150	>150	60.0	36.4	9.0	ND	0.3	0.1	ND	0.13
M1 12	>150	57.6	74.5	>150	28.8	>150	37.4	108.5	ND	0.3	0.7	ND	0.25

a Each value was obtained as technical triplicates, *n* = 3; ND, not determined.

b Kinetic solubility was determined in three solutions at a concentration of 200 μM but at different pH values which represent that of the gastrointestinal tract.

c FaSSIF buffer mimics the fasted state (87).

d Solubility of DHA could not be determined in the buffer solutions because of decomposition.

e Plasma protein binding could not be calculated as DHA was unstable in plasma.

f 11-Aza-artemisinin did not readily dissolve in DMSO, and therefore the calibration standards used in this experiment were prepared in methanol.

It was not possible to measure solubility of DHA at any pH because of decomposition, as noted previously (44, 51). DHA presented high clearance rates in all three microsomal systems, and again in accord with previous observations (43), the extent of plasma protein binding could not be established due to decomposition. The aqueous solubility of artemether was not able to be determined here but is variously recorded to be <1 mg/ml, 12.1 mg/liter at 25°C (69), 0.3 mg/ml (70), or rated as insoluble (71). It also displayed poor metabolic stability with high clearance rates with mouse (MLM), rat (RLM), and human liver microsomes (HLM). This lability coupled with its auto-induction of metabolism is already established (34, 35). The relatively lipophilic artemiside with log *P* of 4.97 and calculated log *P* (ClogP) of 3.98 (Fig. 2) has low aqueous solubility of <2 mg/liter or <5.4 μM at pH 7.2 (51). Here, the compound displayed low aqueous solubility in the FaSSIF buffer at pH 6.5 and phosphate-buffered saline (PBS) buffer at pH 7.4. This suggests that it would be absorbed poorly in the fasted state. At pH 2, the modest increase in solubility to 12.4 μM is ascribed to formation of the more polar conjugate acid, with a calculated pK_a_ of 6.99, arising from protonation of the nitrogen atom of the thiomorpholine ring. Notably, artemiside displayed high intrinsic clearance rates and short half-lives in all three liver microsome systems, predominantly due to its facile metabolism to artemisone as described below. It displayed hepatic extraction ratios in all three systems comparable to those of artemether, again suggesting it would be rapidly metabolized in the liver. The more polar artemisone (log *P* of 2.49, ClogP of 2.08, aqueous solubility at pH 7.2 of 89 mg/liter or 221.6 μM) (51) was appreciably more soluble in the aqueous buffers but displayed high clearance rates and low half-lives in all three liver microsome systems. The artemisone metabolite M1, ClogP 2.79, displayed solubility in the three aqueous buffers on par with that of artemisone but in notable contrast displayed greatly improved half-lives in the liver microsome systems. Interestingly, it exhibited varied metabolic stabilities in the different microsome systems, displaying a moderate clearance rate in MLM, a high clearance rate in RLM, and a very low rate in HLM. The sulfamide 7 is less polar (logP of 3.36, ClogP of 2.29) (51) than artemisone and was reported to possesses good solubility in water at pH 7.2 (294 mg/liter or 811 μM) (51). However, here, while it displayed acceptable solubility at pH 2, it exhibited poor solubility in the other aqueous buffers. It was cleared at a moderate rate by MLM and RLM, although comparatively it had an encouragingly low clearance rate in HLM, indicative of a less facile metabolism. The phenylurea 9 had improved solubility at pH 2 only but displayed very high clearance rates and low half-lives. The arylurea 10 exhibited moderate solubility in all three aqueous buffers and was cleared rapidly in MLM and HLM. 11-Aza-artemisinin 11 displayed the best solubility of all compounds (>144.2 μM) in the three aqueous solutions; as it contains no basic nitrogen atoms, this represents an intrinsic solubility. It also displayed the lowest clearance rates in all three microsome systems with a moderate clearance rate in MLM and a low rate in RLM. Notably, the half-life, clearance rate, and extraction ratio of 11-aza-artemisinin could not be calculated in HLM as it was found to be stable throughout the experiment. This represents the first determination of the metabolic stability of this well-known compound.

The artemisinins presented moderate plasma protein binding as reflected in the unbound fraction ranging from 0.13 to 0.25. The one exception is provided by the arylurea 10, which had the highest plasma protein binding as reflected in the fraction unbound (f_u_) of 0.02.

### Pharmacokinetic properties in mice.

PK properties were determined in healthy male C57BL/6 mice by intravenous (i.v.) (Table 2) and oral (p.o.) administration (Table 3). Calibration curves were used to determine the drug concentrations *in vivo* of the artemisinins from mouse plasma. The calibration curves fitted quadratic regressions over the range of 3.9 × 10^−3 ^μg/ml to 4.0 μg/ml for artemether, artemiside, artemisone, the arylurea, and 11-aza-artemisinin and 1 × 10^−3 ^μg/ml to 4.0 μg/ml for the sulfamide and phenylurea. The accuracy (percentage nominal concentration [Nom]) and precision (percentage of coefficient of variation [CV]) statistics of the low-, medium-, and high-quality controls for artemether, artemiside, the phenylurea, arylurea, and 11-aza-artemisinin were between 72.2 to 125.9% and 0.2 to 11.8%, respectively, and were 71.2% to 111.1% and 0.7 to 14.5% for artemisone and sulfamide, respectively, during sample analysis. Tafenoquine is an 8-aminoquinoline and is prescribed for prophylaxis and treatment of P. vivax infections (72). The PK data of tafenoquine recorded using the murine model described here (73) are included in Tables 2 and 3. It demonstrates a very long half-life, a low intrinsic clearance rate, and a relatively high bioavailability. The properties stand in stark contrast to those of the artemisinins and highlight the notably poor “drug-likeness” (74) of the latter.

**TABLE 2:** Pharmacokinetic parameters calculated from the intravenous administration (i.v.) of the artemisinins to male C57/BL6 mice at a dose of 10 mg/kg (*n* = 3)^*a*^

Compound (i.v.)	*t*_1/2_ (h)	*V* (liter/kg)	CL_tot_ (ml/min/kg)	CL_int_ (ml/min/kg)	AUC (min/μmol/liter)
Artemether 3	1.4 ± 0.2	14.1 ± 1.3	119.7 ± 39.0	855.0	304.4 ± 80
DHA 2 from i.v. artemether 3					155 ± 17
Artemiside 5	1.8 ± 0.5	24.3 ± 9.3	155.0 ± 46.4	673.9	184.9 ± 43
Artemisone 6 from artemiside 5					28.9 ± 1
M1 12 from artemiside 5					13.8 ± 9
Artemisone 6	0.5 ± 0.03	1.9 ± 0.1	42.3 ± 2.1	302.1	577.5 ± 30
M1 12 from artemisone					60.6 ± 36
Sulfamide 7	0.6 ± 0.1	1.63 ± 0.4	32.2 ± 6.2	189.4	864.1 ± 213
Phenylurea 9	1.3 ± 0.1	6.03 ± 0.3	55.9 ± 3.3	399.3	431.9 ± 27
Arylurea 10	1.3 ± 0.3	6.92 ± 2.9	58.9 ± 3.5	2,945.0	315.9 ± 33
11-Aza-artemisinin 11	3.1 ± 0.8	19.4 ± 7.5	75.0 ± 8.9	576.9	462.7 ± 117
Tafenoquine^*b*^	43.7 ± 1.5	2.34 ± 0.0	ND	1.84	2,363 ± 146

a CL_tot_, total plasma clearance; CL_int_, intrinsic plasma clearance; *V*, volume of distribution during elimination phase; AUC, area under the concentration-time curve from 0 h to last; ND, not determined. Mean ± standard deviation (SD) reported.

b Data from reference 73.

**TABLE 3:** Pharmacokinetic parameters calculated from the oral administration (p.o.) of the artemisinins to male C57/BL6 mice at a dose of 50 mg/kg (*n* = 3)^*a*^

Compound (p.o.)	*T*_max_ (h)	*C*_max_ (μM)	AUC (min/μmol/liter)	*F* (%)	Ratio AUC_(0–last)_ metabolite/parent
Artemether 3	0.1 ± 0.0	0.6 ± 0.2	32.3 ± 20	2 ± 0.6	
DHA 2 from artemether 3			77 ± 12		1.9
Artemiside 5	1.0 ± 0.9	0.1 ± 0.01	9.5 ± 2.5	1 ± 0.2	
Artemisone 6 from artemiside 5	1.3 ± 0.8	0.8 ± 0.3	101.5 ± 49		9
M1 12 from artemiside 5	0.3 ± 0.0	0.1 ± 0.02	20.8 ± 8		1.4
Artemisone 6	0.3 ± 0.1	8.0 ± 0.8	922.7 ± 348	34 ± 7.5	
M1 12 from artemisone 6	0.1 ± 0.1	2.1 ± 0.3	173.3 ± 51.7		0.2
Sulfamide 7	0.6 ± 0.2	21.7 ± 1.2	2,562.3 ± 499	59.3 ± 11.6	
Phenylurea 9	0.5 ± 0.0	1.0 ± 0.1	149.5 ± 5.9	6.9 ± 0.3	
Arylurea 10	0.5 ± 0.0	2.3 ± 0.3	276.5 ± 48.4	17.5 ± 3.1	
11-Aza-artemisinin 11	0.5 ± 0.0	2.6 ± 0.01	154.9 ± 10	7.15 ± 0.3	
Tafenoquine^*b*^	9.0 ± 1.0	3.06 ± 0.37	11,368 ± 1,232	55 ± 2	NA

a *C*_max_, maximum concentration; *T*_max_, time to *C*_max_; AUC_0–last_, area under the concentration-time curve from 0 h to last; *F*, oral bioavailability; NA, not applicable. Mean ± SD reported.

b Data from reference 73.

**FIG 3:**
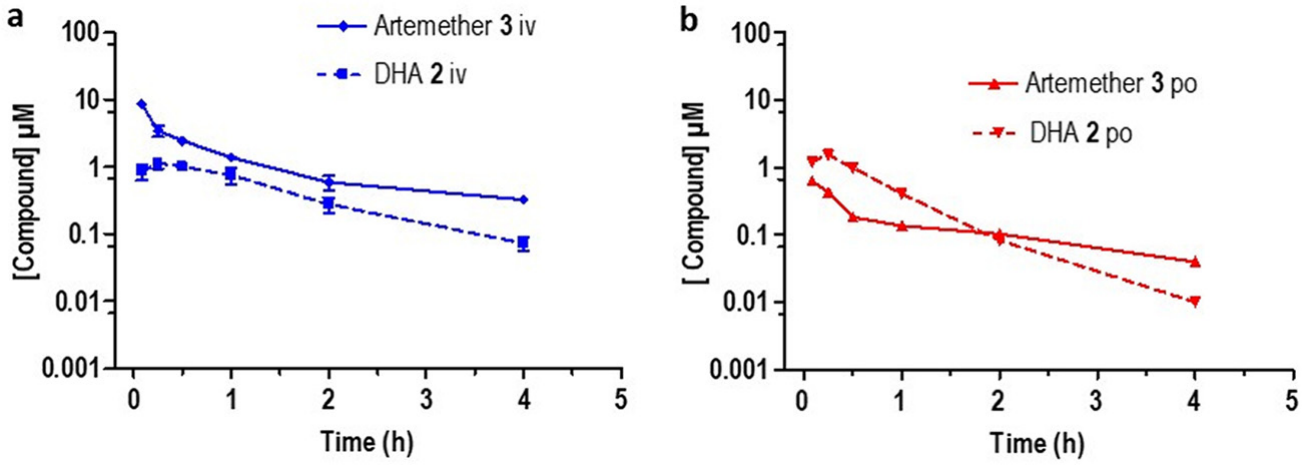
Plasma concentration-time curves for artemether 3 (solid line) and its metabolite DHA 2 formed following (a) intravenous dosing (i.v.) and (b) oral dosing (p.o.). All results are presented as the mean ± standard deviation.

**FIG 4:**
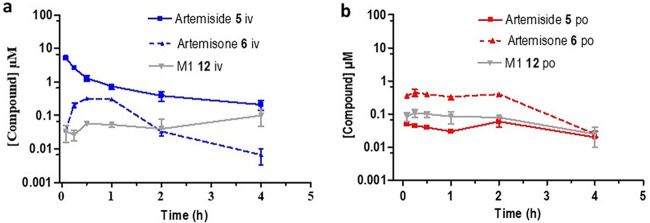
Plasma concentration-time curves for artemiside 5 (solid line) and its metabolites artemisone 6 (dashed line) and M1 12 (solid gray line) formed following (a) intravenous dosing (i.v.) and (b) oral dosing (p.o.). All results are presented as the mean ± standard deviation.

**FIG 5:**
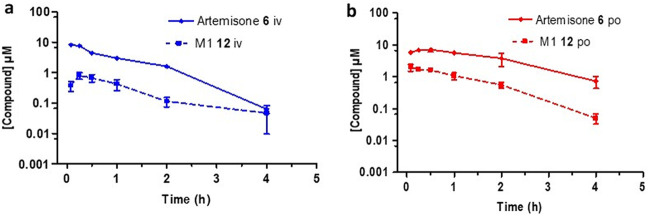
Plasma concentration-time curves for artemisone 6 (solid line) and its metabolite M1 12 (dashed line) formed following (a) intravenous (i.v.) and (b) oral (p.o.) dosing. All results are presented as the mean ± standard deviation.

**FIG 6:**
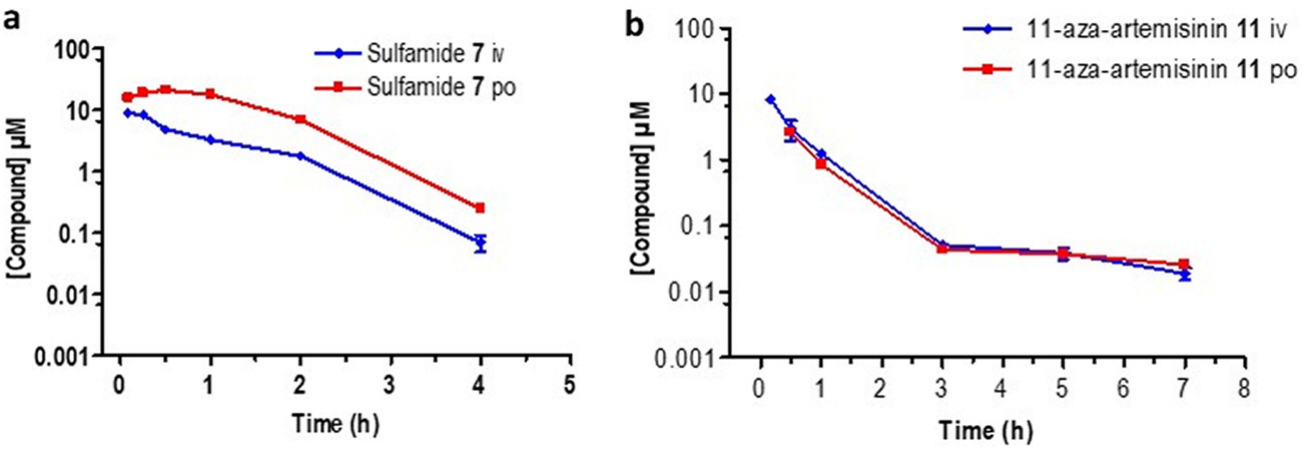
Plasma concentration-time curves for (a) sulfamide 7 and (b) 11-aza-artemisinin 11 following intravenous (i.v., blue line) and oral (p.o., red line) dosing. All results are presented as the mean ± standard deviation.

**FIG 7:**
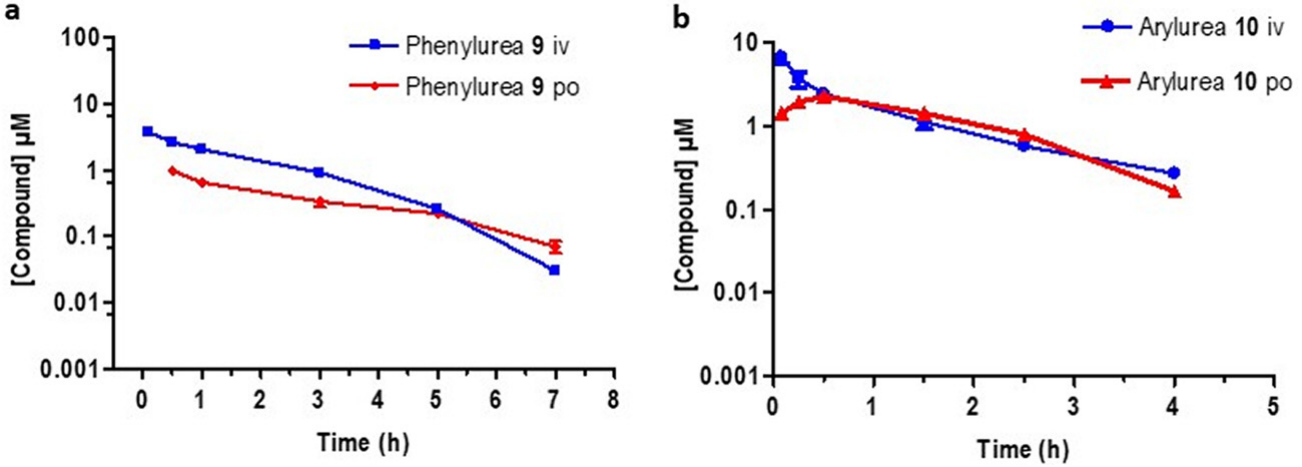
Plasma concentration-time curves for (a) the phenylurea 9 and (b) the arylurea 10 following intravenous (i.v., blue line) and oral (p.o., red line) dosing. All results are presented as the mean ± standard deviation.

The plasma concentration-time curves derived from the PK data are shown in Fig. 3 to 7. Artemether (Fig. 3) exhibited a short half-life (1.4 h) with a high intrinsic clearance rate (855.0 ml/min/kg) and a low bioavailability of 2%. DHA was rapidly formed following i.v. and p.o. dosing (Fig. 3). This underscores the lability of artemether as noted elsewhere (34, 35).

Artemiside displayed a short half-life (1.8 h) as well as a high clearance rate (673.9 ml/min/kg) and a low bioavailability of 1%. Notably, we record here for the first time that it was relatively rapidly metabolized to artemisone and subsequently into the unsaturated metabolite M1 (Fig. 4). Artemisone had the shortest half-life (0.5 h) and a high intrinsic clearance rate (302.1 ml/min/kg), which matches the calculated *in vitro* ADME data (Table 1). Despite this, artemisone displayed PK parameters that were improved relative to those of the other artemisinins (Table 2). It was rapidly converted into the metabolite M1 following an oral dose of 50 mg/kg (Fig. 5) and possessed an improved bioavailability of 32%. This compares quite well with earlier data obtained following a single oral dose of artemisone at each of 3, 10, and 30 mg/kg to female rats (51, 54). Thus, peak plasma concentrations were reached between 15 and 60 min. The corresponding *C*_max_ and elimination half-lives were 35, 230, and 850 μg/liter and 0.18, 0.37, and 0.54 h. Based on intravenous AUC data, the bioavailability of artemisone ranged from 5 to 25%, in rough agreement with that observed here. Also noted earlier was an apparent facile saturation of first pass metabolism based on 4-week toxicological studies in female rats. Importantly, the measured *t*_1/2_ of artemisone in rats was at marked variance with the data obtained from *ex vivo* bioassay studies which suggest that *t*_1/2_ in a primate model is about 2 to 3 h (51). This was also confirmed by *ex vivo* bioassay of human plasma samples taken during phase I studies (54). Thus, bioassay of subject plasma samples showed artemisone equivalent concentrations to be about 2.3-fold higher at *T*_max_ than that measured by liquid chromatography-tandem mass spectrometry (LC/MS-MS) for artemisone. The discrepancy arises because the *ex vivo* bioassay screen measures total activity of artemisone plus the active metabolite M1 and the hydroxylated counterparts in monkey or human plasma.

The sulfamide showed a short half-life (0.6 h) following i.v. or p.o. dosing but revealed promising exposure levels with a bioavailability of 59% (Fig. 6a). It exhibited an intrinsic clearance rate (189.4 ml/min/kg) which was the lowest of the artemisinins evaluated here. 11-Aza-artemisinin (Fig. 6b) displayed the longest half-life (3.1 h) but had a high intrinsic clearance rate (576.9 ml/min/kg) and an oral bioavailability of 7%. This does not correlate with the calculated *in vitro* ADME data which suggest that it is notably stable with a low intrinsic clearance rate (Table 1). The phenylurea 9 (Fig. 7a) had a poor exposure, resulting in a low bioavailability of 6.9%. It also had a short half-life (1.3 h) and a high intrinsic clearance rate (399.3 ml/min/kg). Notably, the arylurea 10 (Fig. 7b), differing from the phenylurea in possessing a fluorine atom at C-4 of the phenyl ring (Fig. 2), presented parameters similar to those of arylurea 9 in possessing a short half-life (1.3 h), a notably rapid clearance (2,945.0 ml/min/kg), and bioavailability of 17.5%.

Importantly, DHA was not detected as a metabolite of either artemiside or artemisone (the latter in accord with previously reported data [51, 54]), the sulfamide, 11-aza-artemisinin, or the phenyl and aryl ureas. The facile metabolism of artemiside to artemisone and thence the metabolite M1 is noteworthy.

## DISCUSSION

The data overall highlight the problems of metabolic stability, poor solubility, and short half-lives of the artemisinins at large. In addition, a comparison with the far more stable and better absorbed tafenoquine emphasizes the lack of drug-likeness and difficulties in applying drug-design precepts to artemisinins (74, 75). However, in comparison with DHA and artemether, the amino-artemisinins do elicit more favorable properties, although within this series in particular, the differences do not enable a clear-cut decision to be made on the “best” amino-artemisinin, with several possible exceptions. Further, any “stop-go” criteria must also incorporate overall cost of production of a particular candidate and especially relative lack of toxicity coupled with efficacy against all stages of the malaria parasite.

The inability to measure the kinetic solubility of DHA or to assess the extent of plasma protein binding because of decomposition reflects its instability (43, 45, 46, 51). However, it was possible to measure clearance rates in the microsomal systems, and these were among the slowest of the artemisinins (Table 1). Artemether displayed low exposure levels as measured by AUC values (Table 3), which is likely due to rapid plasma clearance when administered intravenously. The low bioavailability of artemether (Table 3) reflects its poor absorption and limited systemic exposure. As previously recorded (34, 35), it is rapidly metabolized to DHA; indeed, the transformation is so rapid that artemether is considered a prodrug for DHA (75).

For the first amino-artemisinin artemiside, the PK data resemble those of artemether in terms of rapid plasma clearance and volumes of distribution. The exposure and bioavailability are limited by low aqueous solubility, but they are notably susceptible to microsomal biotransformation (Table 1; Fig. 4) via rapid oxidative conversion of the thiomorpholine sulfide to the sulfone of artemisone. Such a facile sulfide to sulfone biotransformation must proceed via the known sulfoxide artemisox (51) (Fig. 2). An analogous transformation has been mapped out for the anthelmintic drug albendazole incorporating a propylthio group attached to a benzimidazole. This undergoes rapid first pass metabolism via oxidation of the sulfide to the bioactive sulfoxide and then to the sulfone (76). Likewise, metabolism of the antitrypanosomial drug fexinidazole carrying a (4-methylthio)phenoxyl group attached to a nitroimidazole proceeds via way of the corresponding sulfoxide to the sulfone, both more polar metabolites that in contrast to fexinidazole itself elicit blood concentrations above their effective therapeutic doses against the nonapicomplexan parasite Leishmania donovani (77, 78). Thus, while the “prodrug” concept may be applied here to artemiside, the drug is highly active in its own right *in vitro* against *P. falciparum* (Tables S1 and S3). Also, the AUC of artemisone generated *in situ* following oral dosing of artemiside was 9-fold lower than the exposure level achieved upon oral dosing of artemisone itself (Table 3). While this initially suggests that dosing of artemiside does not result in higher levels of artemisone, no account is made here for formation and biotransformation rate of the intermediate artemisox. These aspects, including efficacy data for artemisox, the ramifications for its formation during metabolism of artemiside, and overall effects on bioavailability and plasma levels, are discussed separately.

Artemisone showed PK properties superior to those of artemether. While it possesses a low elimination half-life (Table 2), it has a much higher oral bioavailability (Table 3), reflecting more efficient absorption and slower clearance. These results correlate well with the microsomal stability (Table 1). Improved absorption may be attributed to improved solubility at physiological pH. Artemisone forms the active metabolite M1 bearing unsaturation in the thiomorpholine *S*,*S*-dioxide ring. This metabolite and its hydroxylated derivatives substantially add to overall efficacy of artemisone according to *ex vivo* bioassays of monkey plasma (51, 54). Metabolite M1 elicits solubility in aqueous solutions at the different pH values on par with that of artemisone but, in contrast, possesses substantially greater microsomal half-lives (Table 1). Thus, in comparison to the DHA metabolite formed from artemether or artesunate, M1 is more soluble, is more stable, and possesses excellent *in vitro* activities against asexual blood stages of sensitive and multidrug-resistant *P. falciparum* (see Table S7 in the supplemental material) (63). From a chemical viewpoint, it is noted that while *N*-dealkylation of drugs incorporating six-membered cyclic tertiary amines appears to be the characteristic pathway for metabolism (79, 80), desaturation as observed here is decidedly less prevalent.

The sulfamide showed PK properties that were significantly improved compared to those of artemether, including greatly improved overall systemic exposure, represented by a greater *C*_max_ and a distinctly higher bioavailability (Table 3). Interestingly, the sulfamide had a low volume of distribution and a low clearance rate, which suggest low tissue penetration. The microsomal stability assays suggest that it is not as susceptible to biotransformation as are the other compounds (Tables 2 and 3). It is noted that analogues of the sulfamide have been prepared carrying piperazine-pyridyl substituents (48), and although efficacies are marginally improved, the additional synthetic chemistry efforts cannot now be justified in the face of the accessibility of the sulfamide (51) and its relatively favorable PK profile noted here.

With respect to the phenyl and aryl ureas (Fig. 2), we prepared the latter on the basis that the *p-*fluorine atom would block oxidation of the phenyl ring via arene oxide formation during phase I metabolism, thereby rendering the compound less toxic *in vivo*. Strikingly, however, the arylurea is substantially less toxic *in vitro* against mammalian cell lines, including CHO cells, than is the phenylurea (CHO EC_50_ of 204 μM versus 2.4 μM; Table S1) (48). The arylurea also has solubility that is improved relative to that of the phenylurea at higher pH (Table 1), and both compounds show relatively good bioavailability compared to that of artemether, although it is inferior to that of artemisone (Table 3). The compounds, like artemisone, are rapidly absorbed, as indicated by the respective *T*_max_ values (Table 3).

11-Aza-artemisinin displayed a short half-life following intravenous administration (Table 2), which appears to be contrary to the calculated clearance *in vitro* (Table 1). When administered orally, it was poorly absorbed (Table 3). The compound possesses a secondary amide group that is an efficient H-bond donor-acceptor ensemble (81). Thus, the H-bonding capacity may explain poor absorption *in vivo*; it may not be able to pass via passive diffusion through cell walls. 11-Aza-artemisinin is at a higher oxidation state than is DHA, so it is unable to be metabolized into DHA (82); indeed, the metabolite could not be detected here. While its greatly enhanced thermal and hydrolytic stability with respect to those of other artemisinins have been demonstrated (61), the relative stability evinced here, albeit in *in vitro* screens using microsomes, is encouraging. This well-known compound is prepared in one economic step from artemisinin (61, 82), but it displays relatively mediocre activity *in vitro* against P. falciparum.

New antimalarial compounds should meet one or more target compound profiles (TCP) in order to inhibit the malaria parasite at different stages of its life cycle and thus relieve clinical symptoms, avoid relapse, and block transmission (83). The amino-artemisinins possess potent activities *in vitro* against asexual blood stages of drug-sensitive and -resistant P. falciparum strains, and selected compounds display activities against transmissible gametocyte states that are superior to those of the current clinical artemisinins and indeed of methylene blue (47, 48). These thus have the potential to block transmission, the important TCP5 feature. A substantial advantage of the amino-artemisinins is that none generates DHA as a metabolite but, like the current clinical artemisinins, they do possess relatively short half-lives. The ultimate aim is to use one of these amino-artemisinins in combination with a redox-active drug and a third drug with a different mode of action, both of which possess substantially longer half-lives, in assembling new TACTs for treatment of malaria. In working toward this goal, we have already demonstrated that combinations of artemiside or artemisone with the redox drug methylene blue exert synergistic activity against transmissible-stage P. falciparum gametocytes (47). The high efficacy of methylene blue-based combinations strongly encourages further investigation into combinations involving the amino-artemisinins, redox compounds such as phenoxazines, naphthoquinones, and others, and a third drug with a different mode of action (48).

### Conclusion.

The amino-artemisinins present *in vitro* ADME and *in vivo* PK properties that are similar or improved compared to those of DHA and artemether. Although artemisone is readily metabolized to M1 as reflected in its low elimination half-life, it displays a greater bioavailability (Table 3) that likely is due to improved solubility (Table 1). Its principal metabolite M1 is also soluble, has a much greater microsomal half-life than artemisone, and is highly active against P. falciparum *in vitro*. Although artemiside has low aqueous solubility, its potency against all P. falciparum blood stages, its synergism with methylene blue against transmissible blood-stage parasites (47), and its metabolism to artemisone (and thence the metabolite M1) also favor its continued examination. Of the others, the sulfamide is relatively soluble and, uniquely for an artemisinin, possesses good stability and bioavailability. While the arylurea has inferior solubility and bioavailability, it is easily prepared (48), has relatively low toxicity, and is highly potent against all P. falciparum blood stages. 11-Aza-artemisinin displays good solubility and metabolic stability, but it does have lower efficacy. However, its derivatives are substantially more active against P. falciparum (61, 62) and the apicomplexan parasite Neospora caninum (84), and this aspect coupled with the favorable PK data of the parent encourages their further examination.

## MATERIALS AND METHODS

### Ethics.

All studies and procedures were conducted with prior approval of the animal ethics committee of the University of Cape Town (approval number 013/028) in accordance with the South African National Standards (SANS 10386:2008) for the Care and Use of Animals for Scientific Purposes (85) and guidelines from the Department of Health (86). The use of human plasma was approved by the human research ethics committee of the University of Cape Town (HREC 783/2016).

### Materials.

Preparation of the amino-artemisinins 5 to 10 and 11-aza-artemisinin 11 and assessment of purity are described elsewhere (47, 48, 51, 52, 62). The artemisone metabolite M1 12 was prepared, characterized, and purified as previously described (51). All compounds submitted for screening were ≥95% pure according to high-performance liquid chromatography (HPLC) analyses as previously described (48).

Human whole blood and human plasma were obtained from the Western Province blood transfusion services, Cape Town, South Africa. KH_2_PO_4_ and K_2_HPO_4_ were purchased from Merck (Darmstadt, Germany). Analytical-grade acetonitrile was purchased from Anatech (Johannesburg, South Africa). Analytical-grade dimethyl sulfoxide (DMSO), formic acid, carbamazepine, propranolol hydrochloride, warfarin, procaine hydrochloride, and vinpocetine were obtained from Sigma-Aldrich (St. Louis, MO, USA). NADPH was purchased from Sigma-Aldrich (Steinheim, Germany). Polyethylene glycol (PEG), polypropylene glycol (PPG), and hydroxypropylmethylcellulose (HPMC) were purchased from Sigma-Aldrich (St. Louis, MO, USA). Human, rat, and mouse liver microsomes were obtained from Xenotech (KS, USA). All other reagents were of bioanalytical grade. Water was purified via a Milli-Q purification system (Millipore, Bedford, MA, USA).

For kinetic solubility assays, three solutions with differing pH were prepared. Phosphate-buffered saline (PBS) was prepared by mixing KH_2_PO_4_ (0.2 g), K_2_HPO_4_ (0.115 g), NaCl (0.8 g), and KCl (0.2 mg) in purified water (100 ml). The pH was measured and adjusted using 0.05 M HCl or 0.05 M NaOH to pH 7.4. The pH 2 solution was prepared by adding HCl (37%, 83 μl) to purified water (50 ml). FaSSIF buffer at pH 6.5 was prepared by adding KH_2_PO_4_ (2.042 g) and KCl (3.728 g) to purified water (450 ml) and adjusting the pH to 6.5 using 0.05 M HCl or 0.05 M NaOH as required. Simulated intestinal fluid (SIF) (Biorelevant, London, UK) powder (5.6 mg) was then added to the buffer (25 ml) to complete the process.

For the liquid-liquid extraction of plasma samples, a universal buffer containing the internal standard at pH 8 was utilized. The universal buffer consists of a 1:1 mixture of buffer A comprising CH_3_COOH (5.72 ml), H_3_PO_4_ (6.82 ml), and H_3_BO_3_ (6.183 g) in purified water (1 liter) and buffer B comprising NaOH (20 g) in purified water (1 liter).

### ADME profiling *in vitro*.

**(i) Kinetic solubility.** Duplicate stock solutions of each compound at a concentration of 10 mM in DMSO were prepared in a 96-well plate in PBS at pH 7.4, water at pH 2, and FaSSIF buffer at pH 6.5 to a final concentration of 200 μM (68). These three solutions mimic the pH and conditions of the gastrointestinal tract. As the FaSSIF buffer mimics the fasted state *in vitro*, solubility in this buffer provides a guide as to whether compounds should be dosed with or without meals (87). Calibration standards were prepared for each compound in DMSO at 11, 100, and 220 μM. These standards were used to produce a calibration curve required for calculation of the concentration of each compound in the test solutions. The exception was 11-aza-artemisinin, which proved to be less soluble in DMSO in initial kinetic solubility studies, but it did dissolve in the polar test solutions. The assay was repeated with calibration standards prepared in methanol, and as the standards were within acceptable ranges, the results were considered reliable. Plates were agitated on a plate shaker for 2 h (500 rpm, 22°C). Sample analysis was carried out by liquid chromatography-tandem mass spectrometry (LC-MS/MS) analysis as described in “Sample analysis” below. Peak areas were integrated, and calibration curves were constructed and used to calculate the concentrations of each compound in the three buffers.

**(ii) Metabolic stability in liver microsomes.** The metabolic stability assay was performed in duplicate using 96-deepwell plates. Each compound (0.4 mM) was incubated individually in mouse, rat, and pooled human liver microsomes (0.4 mg/ml) at 37°C in the presence and absence of the cofactor NADPH (1 mM). An aliquot was taken at 0, 5, 10, 30, and 60 min, and the reaction was quenched by adding 300 μl of ice-cold acetonitrile containing the internal standard carbamazepine (0.0236 μg/ml) (88). The test compounds in the supernatant were analyzed by means of LC-MS/MS as described in “Sample analysis” below. The normalized data for each sample were used to calculate the *in vitro* half-life, intrinsic clearance rate, and hepatic extraction ratio from the results of this assay using the following equations (88).Equation 1. Normalized area.

(1) $$\mathrm{Normalized area=}\left(\frac{\mathrm{Peak area of sample at time point}}{\mathrm{Peak area of internal standard}}\right)$$

Equation 2. Percent parent remaining.

(2) %   Parent=Normalized   peak   area   of   sample   at   time pointNormalized   peak   area   of   sample   at  t=0×100

Equation 3. Calculated *t*_1/2_.

(3) $$t_{1/2}=-0.693/\lambda$$

where λ is the slope of the ln (% parent remaining) versus time curve of the compound.Equation 4. CL_int_.

(4) $$CL_{\mathrm{int}}=\left[\frac{0.693}{t_{1/2}(\min)}\right]\times \left[\frac{\mathrm{Volume of incubation}\left(\mathrm{\mu l}\right)}{\mathrm{Microsomal protein}\left(\mathrm{\mu g}\right)}\right]$$

Equation 5. Hepatic extraction ratio.

(5) $$E_{H}=\frac{f_{u}\times CL_{\mathrm{int}}}{\mathrm{QH +}f_{u}\times CL_{\mathrm{int}}}$$

**(iii) Plasma protein binding.** Plasma protein binding was measured using an ultracentrifugation method. The assay was performed using a 96-well microtiter plate with pooled human plasma spiked with the test compound (10 μM). An aliquot was removed and quenched using ice-cold acetonitrile containing the internal standard carbamazepine (0.0236 μg/ml) and placed in the freezer. This served as the total concentration sample. After preincubation at 37°C for 1 h, duplicate aliquots of the spiked plasma were transferred to ultracentrifugation tubes and ultracentrifuged for 4 h (216,937 × *g*, 37°C, Beckman Optima L-80XP). Analyte concentration of all compounds and sample types were determined by means of LC-MS/MS as described in “Sample analysis” below.

### Pharmacokinetics *in vivo*.

**(i) Animals.** Healthy male C57/BL6 mice each weighing approximately 25 g were used. The mice maintained at the University of Cape Town animal facility were housed in 27 by 21 by 18 cm cages, under controlled environmental conditions at 22 ± 2°C and a 12-h light/dark cycle. Humidity was monitored and ranged between 55 and 70%. Food and water were available *ad libitum*. Mice were acclimatized to their environment for 7 days before experiments were initiated.

**(ii) Oral drug administration.** Each of artemether, the amino-artemisinins 5 to 10, and 11-aza-artemisinin 11 (12.5 mg) was dissolved in 0.2 ml DMSO, to which 1.8 ml 0.5% (wt/vol) HPMC in water was then added. Compounds were administered orally via gavage at a dose of 50 mg/kg (*n* = 3). Dosing volumes were calculated based on the weight of each mouse in the respective group. The dosing volumes ranged between 160 μl and 200 μl, ensuring that the dose remained at 50 mg/kg. Blood samples for amino-artemisinins 5 to 8 and 10 were collected via tail-bleeding predose and at 0.08, 0.25, 0.5, 1, 2, and 4 h. Blood samples for the phenylurea 9 and 11-aza-artemisinin 11 were collected at 0.08, 0.5, 1, 2, 4, 8, and 24 h postdose. Samples were placed in 0.5 ml lithium heparin micro vials to prevent blood coagulation. Artemether and the amino-artemisinins 5 to 8 and 10 were expected to display the characteristic short half-lives of the artemisinins, and therefore it was more important to map the initial exposure over 4 h. The phenylurea 9 and the 11-aza-artemisinin 11 were predicted to have longer half-lives and were sampled over a greater time period. The samples were immediately centrifuged at 5,590 × *g* for 5 min and the plasma was transferred to new microvials. The plasma samples were stored at −80°C.

**(iii) Intravenous drug administration.** For the intravenous formulations, each of artemether, the amino-artemisinins 5 to 10, and 11-aza-artemisinin 11 (2.5 mg) was dissolved in 1 ml DMSO/PEG/PPG (1:3:6, vol/vol). DMSO was added first, followed by PEG and then PPG. The resulting solutions in the mixture of DMSO, PEG, and PPG (1:3:6, vol/vol) were administered intravenously at a dose of 10 mg/kg (*n* = 3) via injection into the dorsal penile vein. Mice were anesthetized with an intraperitoneal injection of a saline ketamine/xylazine solution. This consisted of 1 ml ketamine (100 mg/ml), 0.5 ml xylazine (20 mg/ml), and 8.5 ml PBS solution to make a total volume of 10 ml. Dosage of anesthetic preparation for mice was 0.8 ml/10 g body weight; this amounts to a dose of 80 mg/kg ketamine and 10 mg/kg xylazine. The total volume of administration was dose adjusted to 10 mg/kg with an approximate volume of 80 μl, according to the weight of each animal on the morning of dosing. Blood samples for the amino-artemisinins 5 to 8 and 10 were collected via tail-bleeding predose and at 0.08, 0.25, 0.5, 1, 2, and 4 h, and blood samples for the phenylurea 9 and 11-aza-artemisinin 11 were collected at 0.08, 0.5, 1, 2, 4, 8, and 24 h postdose. Samples were all placed in 0.5 ml lithium heparin microvials to prevent blood coagulation. The samples were immediately centrifuged at 5,590 × *g* for 5 min and the plasma was transferred to new microvials. The plasma samples were stored at −80°C.

### Sample analysis.

Concentrations of the kinetic solubility, metabolic stability, plasma stability, plasma protein binding samples, and plasma samples from murine studies were determined using quantitative LC-MS/MS assays.

**Sample extraction.** For sample extraction from the kinetic solubility, metabolic stability, plasma stability, and plasma protein binding studies, each compound was extracted from 200 μl of acetonitrile by adding 350 μl ethyl acetate, which contained the internal standard carbamazepine, and vortexing for 1 min followed by centrifugation at 5,590 × *g* for 5 min. The organic layer was then transferred and evaporated under a gentle stream of nitrogen at room temperature for 30 min. The dried samples were reconstituted in mobile phase, and a 5 μl sample was injected for LC-MS/MS analysis. The only exception was the arylurea 10, for which no liquid-liquid extraction was conducted; instead, the 200 μl acetonitrile solution was evaporated under a gentle stream of nitrogen at room temperature. The dried sample was reconstituted in the mobile phase, and a 5 μl sample was injected for LC-MS/MS analysis. The plasma samples for each compound from the *in vivo* studies were extracted by mixing 350 μl ethyl acetate with 15 μl of plasma sample and 35 μl of universal buffer containing internal standard at pH 8. The samples were vortexed for 1 min and centrifuged for 5 min, and the organic layer was then transferred and evaporated under a gentle stream of nitrogen at room temperature for 30 min. The dried samples were each reconstituted in the mobile phase, and a 5 μl sample was injected for LC-MS/MS analysis.

**LC-MS/MS analysis.** For all the artemisinins except the arylurea 10, gradient chromatography was performed on a Phenomenex (Torrance, CA, USA) Luna PFP(2) analytical column (100 Å, 5 μm, 2 by 50 mm) and an AB Sciex API 5500 mass spectrometer which was operated at unit resolution in the multiple reaction monitoring (MRM) mode. The HPLC method used was a gradient method with 5 mM ammonium acetate and 0.1% acetic acid in water as the aqueous phase and a mixture of acetonitrile and methanol (1:1 vol/vol) containing 5% of the aqueous phase as the organic phase (89). The arylurea 10 was analyzed using a Phenomenex Gemini NX-C18 (5 μm, 2.1 mm by 50 mm) column with an AB Sciex 3200 QTRAP mass spectrometer operated at unit resolution in the MRM mode. The HPLC method used was a gradient method with 5 mM ammonium acetate and 0.1% acetic acid in water as the aqueous phase and a mixture of acetonitrile and methanol (1:1 vol/vol). The transitions of the molecular ions were monitored, as listed in Table S8 in the supporting information. The calibration range was between 0.002 μg/ml and 6.250 μg/ml. Data acquisition and evaluation were performed using Analyst 1.6.2 software (Applied Biosystems, Foster City, CA, USA). The formation of the putative metabolite DHA from the amino-artemisinins and the metabolite M1 of artemisone were evaluated in these mice PK studies; the MRM transitions of DHA and M1 (Table S8) were thus included in the LC-MS/MS assays. Data for the artemether product ion are consistent with those presented elsewhere (35).

**Data analysis.** Drug concentration versus time plots for each compound were used to determine maximal drug concentration *C*_max_, time *T*_max_ to reach *C*_max_, elimination half-life *t*_1/2_, and the area under the concentration-time curve from time zero to last, AUC_0–last_. From these values, the PK parameters clearance (CL), volume of distribution (*V*), and oral bioavailability (*F*) could be determined using the noncompartmental analysis software PK Solutions version 2.0, Summit Research Services, Montrose, USA. The area under the plasma concentration curve up to the last time point AUC_0–last_ was calculated by using the trapezoidal rule (90).
