# Supplementary material for: Toward New Transmission-Blocking Combination Therapies: Pharmacokinetics of 10-Amino-Artemisinins and 11-Aza-Artemisinin and Comparison with Dihydroartemisinin and Artemether
Source: Antimicrob Agents Chemother. 2021 Jul 16;65(8):e00990-21. doi: 10.1128/AAC.00990-21 (PMC8284440; doi:10.1128/AAC.00990-21)
Supplement: Supplemental file 1 — Supplemental material. Download AAC00990-21_Supp_S1_seq2.pdf, PDF file, 0.3 MB [file aac00990-21_supp_s1_seq2.pdf]

Towards new transmission-blocking combination therapies - pharmacokinetics of 10-amino-artemisinins and 11-aza-artemisinin, and comparison with DHA and artemether

Running Title: New Artemisinin Combination Therapies and Pharmacokinetics of Amino-artemisinins.

Daniel J Watson,[1] Lizahn Laing,[1] Liezl Gibhard,[2] Ho Ning Wong,[3] Richard K. Haynes,[3] Lubbe Wiesner[1]

[1]Division of Clinical Pharmacology, Department of Medicine, University of Cape Town, Observatory, 7925, South Africa

[2]H3D, Department of Chemistry, University of Cape Town, South Africa

[3]Centre of Excellence for Pharmaceutical Sciences, Faculty of Health Sciences, North-West University, Potchefstroom, 2520, South Africa.

Supporting Information

1. Efficacy data from references 1,2,3,4,5

Table S1: Activities *in vitro* against chloroquine-sensitive and multidrug resistant asexual blood stage *P. falciparum* and cytotoxicities of amino-artemisinins (refs. 1 and 2).[a]

[a]Compound	IC_{50} nM[b]					*Cytotoxicity* EC_{50}[c]	
	NF54	K1	RI[e]	W2	RI[f]	CHO µM	SI[g]
Chloroquine	10.0±3.0	154±14	15.4	233±49	23.3	ND	ND
Methylene Blue	5.0±0.8	6.45±0.30	1.29	5.13±0.31	1.03	52.6±4.5	175333
DHA **2**	2.51±0.19	1.51±0.33	0.6	1.74±0.22	0.7	25.2	10039
Artemiside **5**	1.11±0.17	1.6±0.4	1.47	1.75±0.27	1.58	>271	>45166
Artemisone **6**	1.2±0.4	1.01±0.19	0.85	1.6±0.4	1.36	>249	>83000
Sulfamide **7**	3±1	1.78±0.26	0.56	2.04±0.11	0.64	56.0±4.6	51376
Arylamine **8**	1.3±0.6	0.64±0.10	0.48	3±1	2.55	2.9±1.4	371
Phenylurea **9**	4.7±1.5	2.9±0.6	0.61	1.7±0.5	0.36	2.4±1.0	1846
Arylurea **10**	1.3±0.1	0.85±0.1	0.65	1.16±0.17	0.9	204	156923

[a]Structures in Figs. 1,2; *P. falciparum* NF54 CQ sensitive;K1: CQ, pyrimethamine, mefloquine, cycloguanil resistant; W2: CQ, quinine, pyrimethamine, cycloguanil resistant; [b]Results for proliferative (SYBR Green I) assays are from three independent biological replicates, each performed as technical triplicates; ± SEM; [c]Cytotoxicity studies using the MTT assay against Chinese hamster ovary (CHO) mammalian cells were performed for a single independent biological repeat, each performed as technical duplicates/triplicates, ±SD; [d]Resistance index (RI) = IC_{50} Dd2/IC_{50} NF54; [e]RI = IC_{50} K1/IC_{50} NF54; [f]IC_{50} Dd2/IC_{50} NF54; [g]Selectivity index (SI) = EC_{50} CHO/IC_{50} NF54 proliferative assay.

Table S2: *In vitro* biological data for artemisinins, 11-aza-artemisinin **11**. Results are representative of three independent biological replicates, each performed as technical triplicates (ref. 4).

	Antimalarial activities IC_{50} nM ± SEM			Resistance Index		Cytotoxicities IC_{50} (µM)	Selectivity Index
Cpd	NF54	K1	W2	RI[a]	RI[b]	WI-38 HFLF[c]	SI[d]
DHA **2**	0.57 ± 0.01	0.8 ± 0.5	0.37 ± 0.11	1.40	0.65	ND	ND
Artemether **3**	1.86 ± 0.17	2.64 ± 0.18	6.7 ± 0.6	1.41	3.60	ND	ND
Artesunate **4**	3 ± 0.29	3.26 ± 0.2	2.4 ± 0.2	1.09	0.8	ND	ND
11-azaartemisinin **11**	10.48 ± 1.36	9.44 ± 2.93	6.02 ± 1.81	0.90	0.57	>100	>9542

[a]Resistance index (RI) = IC_{50} K1/IC_{50} NF54. [b]Resistance index (RI) = IC_{50} W2/IC_{50} NF54. [c]Cytotoxicity data. [d]Selectivity index (SI) = IC_{50} WI-38/IC_{50} NF45. ND = not determined

Table S3: Activities of amino-artemisinins *in vitro* against early and late blood stage *P. falciparum* NF54 gametocytes (refs. 1 and 2)[a]

[a]Compound	[b]Early Stage (EG, Luc 48 h) IC_{50} nM	[c]Late Stage (LG, Luc 72 h) IC_{50} nM	Fold change preference ratio: EG to LG	Fold change preference ratio: LG to EG
Methylene Blue	95.0±11.3	143.0±16.7	1.5	0.7
DHA **2**	43.0±3.9	33.66±1.98	0.78	1.3
Artemiside **5**	16.4±1.0	1.5±0.5	0.09	10.9
Artemisone **6**	1.94±0.11	42.4±3.3	21.9	0.05
Sulfamide **7**	15.0±2.0	419.4±59.5	28.0	0.04
Arylamine **8**	38.2±9.0	16.42±6.38	0.4	2.3
Phenylurea **9**	83±2	1.70±0.99	0.02	48.8
Arylurea **10**	36.0±10.2	17.0±7.0	0.47	2.59

[a]Structures in Figs. 1, 2; IC_{50} values against [b]early stage (>90% stage I-III) and [c]late stage (>90% stage IV and V) gametocytes determined using the luciferase based assay against the Luc reporter cell line. Results are representative of the mean of three independent biological replicates, performed as technical triplicates, ± SEM. Data are from a 72-h incubation period.

Table S4: Activities *in vitro* for artemisinins, azaartemisinin **11** against early stage (I-III) and late stage (IV-V) *P. falciparum* NF54gametocytes (ref. 4).[a]

	Early stage	Late stage		Stage specificity[b]
Compound	IC_{50} (nM) ± SEM (n = 3) (Luciferase 48 h)	IC_{50} (nM) (n = 1) (Luciferase 72 h)	IC_{50} (nM) (n = 1) (pLDH 72 + 72 h)	[pLDH IC_{50} (nM)/ NF54 IC_{50} (nM)]
Artemether **3**	37.74 ± 2.08	223.9	ND	122.35[b]
Artesunate **4**	62.83 ± 3.14	171.0	ND	57.0[b]
11-aza-artemisinin **11**	170.4 ± 19.3	166.1	622.2	59.37

[a]Results against the early stages are from three independent biological replicates, performed as technical triplicates (± SEM), and for the late stages as a single independent biological replicate, performed as technical triplicates;[b] Stage specificity for artemether and artesunate: luciferase 72 h IC_{50} (nM)/NF54 IC_{50} (nM); ND = not determined

Table S5: *In vitro* activities of selected amino-artemisinins against liver stage *P. berghei* sporozoites and cytotoxicities (ref. 2)[a]

Compound	IC_{50} nM	Maximum inhibition % (Conc. µM)	Cytotoxicity EC_{50}[b]	
	P. berghei sporozoites		HepG2 µM	SI
Atovaquone	2.515±0.997	94.85±2.76 (0.5)	> 0.25	> 100
Puromycin	22.7±4.525	110±4.24 (5)	0.117	5.15
Artemether **3**	>10^4	49.4 (10)	ND	ND
Artemiside **5**	81.3±9.616	99.05±1.34 (5)	> 25.0	> 308
Artemisone **6**	28.3±01.273	93.35±1.76 (10)	> 50.0	> 1767
Arylurea **10**	105.5±6.363	94.85±2.76 (10)	5.16	48.9

[a] Structures in Figs. 1, 2; luciferase-expressing *P. berghei* ANKA GFP-Luc-SM_{con} sporozoites were allowed to invade HepG2 cells and luciferase activity was measured after 48 h; data ± SD from biological duplicate and technical quadruplicate measurements. SI = EC_{50} HepG2/IC_{50} *P. berghei* sporozoites.

Table S6*: In vitro* antimalarial activities of amino-artemisinins against *P. falciparum* asexual blood stage artemisinin-resistant clones carrying the *Pf*KI3 C580Y mutation as determined with the T_0 [^{3}H]-hypoxanthine drug susceptibility assay (ref. 2).

**Compound** [a]	**W2**		**ARC08-22 (4G)** (48 h lc)[b]			**PL08-009 (5C)** (36 h lc)[b]		
	IC_{50} nM	IC_{90} nM	IC_{50} nM	IC_{90} nM	RI[b]	IC_{50} nM	IC_{90} nM	RI[c]
DHA **2**	4.58±2.54	10.30±5.76	6.68±0.61	15.40±1.39	1.5	6.41±1.54	10.73±3.81	1.4
Artemiside **5**	2.21±0.42	4.28±0.25	2.43±0.13	4.85±0.09	1.1	0.29±0.03	0.43±0.03	0.1
Artemisone **6**	1.69±0.36	3.42±0.45	1.62±0.19	3.38±0.28	1.0	0.27±0.05	0.43±0.07	0.2
Sulfamide **7**	4.87±0.59	7.53±0.23	4.76±0.25	7.55±0.26	1.0	1.81±0.20	2.38±0.06	0.4
Arylamine **8**	4.76±0.38	8.51±0.34	5.51±0.59	10.92±0.95	1.2	15.73±0.72	22.57±1.00	3.3
Phenylurea **9**	0.12±0.03	0.20±0.03	0.19±0.02	0.36±0.03	1.6	4.71±0.60	6.45±1.81	39.3

[a] Structures in Figs. 1, 2; [b]lc = life cycle (h); [c]RI = IC_{50} for ARC08-22(4G)/IC_{50} for W2; [d]RI = IC_{50} for PL08-09 (5C)/IC_{50} for W2; Results are the mean of three independent biological replicates, performed as technical triplicates, ± SEM.

Table S7: *In vitro* antimalarial activities for chloroquine CQ, mefloquine MFQ, atovaquone, DHA **2**, artesunate **3**, artemisone **6**, metabolite M1 **12** (ref. 5).

	Antimalarial activities IC_{50} nM ± SEM					
Compound	D6	W2	7G8	TM93-C1088	TM91-C235	TM-C2B
Chloroquine CQ	16 ± 2	195 ± 70	84 ± 18	360 ± 38	70 ± 12	95 ± 23
Mefloquine MFQ	ND	ND	5.4 ± 1.7	16 ± 5.0	107 ± 41	130 ± 51
Atovaquone	ND	ND	3.1±0.9	18,830 ± 5,102	2.2 ± 0.7	31,850 ± 6,833
DHA **2**	1.7 ± 0.4	2.2 ± 0.8	1.3 ± 0.2	1.4 ± 0.4	2.3 ± 0.7	2.0 ± 0.7
Artesunate **3**	ND	3.0 ± 1.6	1.5 ± 0.2	1.4 ± 0.1	2.9 ± 1.4	2.5 ± 0.5
Artemisone **6**	1.0 ± 0.4	1.3 ± 0.5	0.8 ± 0.1	0.7 ± 0.2	1.1 ± 0.5	1.1 ± 0.4
Metabolite M1 **12**	4.7 ± 0.2	6.6 ± 0.4	2.6 ± 0.8	2.5 ± 0.4	5.0 ± 0.6	8.6 ± 8.2

[a]D6: CQ sensitive; W2 and 7G8: CQ resistant; TM90-C2B and TM93-C1088: atovaquone and CQ resistant; TM91-C235: CQ and mefloquine resistant; values represent the mean ± SD from three independent experiments carried out in triplicate.

2. Mass spectroscopic transitions of the precursor and product ions

Table S8: MS/MS masses for the artemisinins evaluated by LC-MS/MS

Compound	Precursor ion (*m/z*)	Product ion (*m/z*)
DHA **2**	302.1[a]	284.0
Artemether **3**	316.2[a,b]	267.1
Artemiside **5**	370.2	163.1
Artemisone **6**	402.1	163.0
Sulfamide **7**	380.0[a]	163.1
Phenylurea **9**	472.1	206.1
Arylurea **10**	490.3	224.2
11-Aza-artemisinin **11**	282.1	151.0
M1 **12**	400.2	267.0

[a]Ions were measured as ammonium adducts [$M+NH_4^+$]; all other ions were measured as protonated ions [$M+H^+$]. [b]Data for artemether product ion is consistent with that reported elsewhere (ref. 6)

1 Coertzen, D., Reader, J., van der Watt, M., Nondaba, S.H., Gibhard, L., Wiesner, L., et al. (2018) Artemisone and artemiside - potent pan-reactive antimalarial agents that also synergize redox imbalance in *P. falciparum* transmissible gametocyte stages. *Antimicrob. Agents Chemother*. 62, e02214-17; doi:10.1128/AAC.02214-17.

2 Wong, H.N., Padín-Irizarry, V., van der Watt, M.E., Reader, J., Liebenberg, W., Wiesner, L. et al.(2020) Optimal 10-aminoartemisinins with potent transmission-blocking capabilities for new artemisinin combination therapies – activities against blood stage *P. falciparum* Including *Pf*KI3 C580Y mutants and liver stage *P. berghei* parasites. *Front. Chem.* 7, 901. doi: 10.3389/fchem.2019.00901.

3 Haynes, R.K., Wong, H.-N., Lee, K.W., Lung, C.-M., Shek, L.-Y., Williams, I.D., Croft, S.L., Vivas, L., Rattray, L., Stewart. L. (2007) Preparation of *N*-sulfonyl and -carbonyl-11-azaartemisinins with greatly enhanced thermal stabilities: in vitro antimalarial activities. *ChemMedChem* 2, 1464-1479. doi: 10.1002/cmdc.200700065.

4 Harmse, R., Coertzen, D., Wong, H.N., Smit, F.J., van der Watt, M.E., Reader, J., Nondaba, S.H., Birkholtz, L.-M., Haynes, R.K., N'Da, D.D. (2017) Activities of 11-azaartemisinin and *N*-sulfonyl derivatives against asexual and transmissible malaria parasites. *ChemMedChem* 12, 2086-2093; doi: 10.1002/cmdc.201700599.

[5] Grobler, L., Chavchich, M., Haynes, R.K., Edstein, M.D., Grobler, A.F. (2014). Assessment of the induction of dormant ring stages in *Plasmodium falciparum* parasites by artemisone and artemisone entrapped in Pheroid vesicles *in vitro*. *Antimicrob. Agents Chemother.* 7579-7582. doi: 10.1128/AAC.02707-14.

[6] Liu, T., Du, F., Zhu, F., Xing, J. (2014) Metabolite identification of artemether by data-dependent accurate mass spectrometric analysis using an LTQ-Orbitrap hybrid mass spectrometer in combination with the online hydrogen/deuterium exchange technique. *Rapid Commun. Mass Spectrom.* 25, 3303-3313. doi: 10.1002/rcm.5214.
